# Natural Killer Cells Regulate Th17 Cells After Autologous Hematopoietic Stem Cell Transplantation for Relapsing Remitting Multiple Sclerosis

**DOI:** 10.3389/fimmu.2018.00834

**Published:** 2018-05-07

**Authors:** Peter J. Darlington, Brandon Stopnicki, Tarik Touil, Jean-Sebastien Doucet, Lama Fawaz, Morgan E. Roberts, Marie-Noëlle Boivin, Nathalie Arbour, Mark S. Freedman, Harold L. Atkins, Amit Bar-Or

**Affiliations:** ^1^Departments of Exercise Science and Biology, PERFORM Centre, Concordia University, Montreal, QC, Canada; ^2^Neuroimmunology Unit, McGill University and Montreal Neurological Institute, Montreal, QC, Canada; ^3^Clinical Biological Imaging and Genetic Repository, McGill University, Montreal, QC, Canada; ^4^Department of Neurosciences, Université de Montréal, Centre de Recherche du Centre Hospitalier de l’Université de Montréal (CRCHUM), Montréal, QC, Canada; ^5^Department of Medicine, Ottawa Hospital Research Institute, University of Ottawa, Ottawa, ON, Canada; ^6^Blood and Marrow Transplant Program, Ottawa General Hospital, Ottawa, ON, Canada; ^7^Center for Neuroinflammation and Experimental Therapeutics and Department of Neurology, Perelman School of Medicine, University of Pennsylvania, Philadelphia, PA, United States

**Keywords:** multiple sclerosis, natural killer cells, aHSCT, Th17 cells, NKG2D, CD58

## Abstract

In autoimmunity, the balance of different helper T (Th) cell subsets can influence the tissue damage caused by autoreactive T cells. Pro-inflammatory Th1 and Th17 T cells are implicated as mediators of several human autoimmune conditions such as multiple sclerosis (MS). Autologous hematopoietic stem cell transplantation (aHSCT) has been tested in phase 2 clinical trials for MS patients with aggressive disease. Abrogation of new clinical relapses and brain lesions can be seen after ablative aHSCT, accompanied by significant reductions in Th17, but not Th1, cell populations and activity. The cause of this selective decrease in Th17 cell responses following ablative aHSCT is not completely understood. We identified an increase in the kinetics of natural killer (NK) cell reconstitution, relative to CD4^+^ T cells, in MS patients post-aHSCT, resulting in an increased NK cell:CD4^+^ T cell ratio that correlated with the degree of decrease in Th17 responses. *Ex vivo* removal of NK cells from post-aHSCT peripheral blood mononuclear cells resulted in higher Th17 cell responses, indicating that NK cells can regulate Th17 activity. NK cells were also found to be cytotoxic to memory Th17 cells, and this toxicity is mediated through NKG2D-dependent necrosis. Surprisingly, NK cells induced memory T cells to secrete more IL-17A. This was preceded by an early rise in T cell expression of *RORC* and *IL17A* mRNA, and could be blocked with neutralizing antibodies against CD58, a costimulatory receptor expressed on NK cells. Thus, NK cells provide initial co-stimulation that supports the induction of a Th17 response, followed by NKG2D-dependent cytotoxicity that limits these cells. Together these data suggest that rapid reconstitution of NK cells following aHSCT contribute to the suppression of the re-emergence of Th17 cells. This highlights the importance of NK cells in shaping the reconstituting immune system following aHSCT in MS patients.

## Introduction

Pro-inflammatory helper T (Th) cells contribute to disease activity in several autoimmune inflammatory conditions including multiple sclerosis (MS). In MS, Th1 and Th17 subsets have been considered pathogenic, while Th2 and regulatory T cell subsets may limit disease activity (1). Th17 cells express IL-17A and the transcription factor RORγt. These cells, including those co-expressing IL-17 and IFN-γ, have the capacity to disrupt the blood–brain barrier and directly injure neurons, and therefore have been of particular interest in the study of MS (2, 3).

Natural killer (NK) cells are part of the innate immune system, specialized in killing virus-infected cells and tumors through several mechanisms and receptors including NKG2D-dependent cytotoxicity. Infected or stressed cells express ligands for NKG2D including MICA, MICB, and the ULBP ligands. In addition, NK cells can regulate adaptive immunity (4, 5). There are at least two major subtypes of NK cells in humans referred to as CD56^bright^ and CD56^dim^ NK cells, circulating in blood and located within tissues (6, 7). They are discernable by flow cytometry based on their relative intensity of CD56 expression. In blood, about 90% of NK cells are expected to be CD56^dim^ NK cells, this proportion varies for NK cells within tissues (6, 7). The traditional view was that CD56^dim^ NK cells are cytotoxic while CD56^bright^ NK cells played more of a regulatory role, however, CD56^bright^ NK cells can acquire cytotoxic properties, and both types can produce cytokines (8). NK cells can also be subdivided based on CD16, a low affinity Fc receptor. CD56^dim^ NK cells are predominantly CD16^+^ with only a minor proportion that are CD56^dim^CD16^−^, and CD56^bright^ NK cells are mostly CD56^bright^CD16^−^ with a minor population expressing intermediate levels of CD16, referred to as CD56^bright^CD16^dim^ (7).

Several lines of evidence indicate that NK cells play a protective role in autoimmunity. Depletion or removal of NK cells exacerbates collagen-induced arthritis (9, 10), a transfer model of colitis (11), and experimental autoimmune encephalomyelitis (12–15). Furthermore, dysregulation or impairment of NK cells has been linked to MS relapses and MRI lesions (16–24). The mechanism by which NK cell dysfunction contributes to MS is still unclear, but may include loss of cytotoxic capacity against autologous T cells (25). Following activation, effector T cells are sensitized to NKG2D-dependent cytotoxicity by upregulating NKG2D ligands (26–29). Given their regulatory capacity, NK cells are potential therapeutic targets in autoimmune diseases including MS. Several MS therapeutics limit MS disease activity and concomitantly expand NK CD56^bright^ cells, including daclizumab (30), alemtuzumab (31), dimethylfumarate (32), and IFN-β (33). NK cells are also capable of injuring astrocytes and neurons (34) and promoting demyelination (35). Their overall contribution to autoimmunity is thus a balance of immune-regulatory and pro-inflammatory actions.

Autologous hematopoietic stem cell transplantation (aHSCT) as a treatment for relapsing remitting MS, has been tested in several clinical trials and has been found to be effective at inducing prolonged disease stabilization (36). In a Canadian multi-center phase 2 clinical trial, relapsing remitting MS patients with aggressive disease received immunoablative chemotherapy followed by CD34^+^ aHSCT. In this open label study, treatment resulted in complete abrogation of all clinical relapses and there were no new brain MRI lesions with follow-up now exceeding 13 years (37–39). The concept behind aHSCT therapy in MS is to ablate the existing pathogenic immune cells with the induction chemotherapy regimen and then allow reconstitution of a newly derived, non-pathogenic, autologous immune repertoire by the transplanted stem cells. Studies carried out following aHSCT demonstrated that central nervous system-reactive Th1 cells re-emerged to levels that were indistinguishable from those documented before the treatment. By contrast, Th17 responses, including IL-17 secretion, were much reduced in the reconstituted immune system following aHSCT (40).

Given the importance of NK cells in MS as well as their capacity to limit effector T cell responses, we questioned whether the decrease in Th17 responses after aHSCT, occurs as a result of NK cell-mediated restriction of the generation and/or maintenance of Th17 cells in the early reconstituting immune repertoire. NK cells are known to reconstitute rapidly following allogeneic stem cell transplantation for leukemia (41). Furthermore, a recent study on immunoablative and stem cell reconstitution in MS found a transient increase in NK cells and lower Th17 cell responses post stem cell transplantation (42). In this study, we demonstrated that NK cells were elevated in aHSCT samples.

## Materials and Methods

### Collection of aHSCT Blood Samples From MS Patients

Multiple sclerosis patients were serially enrolled in the immune monitoring sub study as part of the Canadian Collaborative MS/BMT Study (registered at ClinicalTrials.gov, NCT01099930), following informed consent as approved by the institutional ethics review boards. aHSCT therapy was performed on a cohort of MS patients as previously described (39). In brief, patients with aggressive disease who were refractory to drug treatments underwent autologous hematopoietic stem cell mobilization using cyclophosphamide and filgrastim followed by stem cell graft collection by peripheral vein leukopheresis. Immune cells were depleted from the graft using CD34 immunomagnetic selection and the graft was cryopreserved. High dose busulfan, cyclophosphamide, and antithymocyte globulin were administered for immune ablation followed by infusion of the thawed CD34 autologous hematopoietic stem cell graft. The detailed protocol, patient characteristics, and clinical results are published elsewhere (39). Venous blood was sampled from MS patients within 2 months before aHSCT, then at 3 weeks, 3 months, 12 months, and 21 months following aHSCT. A portion of the blood sample was analyzed by flow cytometry with antibodies for CD3 (UCHT-1), CD56 (B159), CD4 (RPA-T4), CD8 (RPA-T8) (from BD Bioscience; Mississauga, ON, Canada), and CD58 (TS2/9; eBioscience). The remaining blood sample was processed into peripheral blood mononuclear cell (PBMC) using Ficoll, aliquoted, and cryopreserved in fetal bovine serum with 10% dimethyl sulfoxide in a cryovial stored in liquid nitrogen.

### Analysis of aHSCT Blood Samples

After completion of the clinical trial, selected aliquots were thawed in batches and used for NK cell bright dim analysis, the Th17 assay, and the CD56-depletion experiment. NK bright and dim were assessed by staining samples for CD3-APC (UCHT-1), CD56-PE (B159), and CD16-FITC (CLB/FcGran1) all from BD Bioscience, then by flow cytometry determining the two different populations, which were visible on the CD3 and CD56 plots as the CD3^−^CD56^bright^ or CD3^−^CD56^dim^ populations, and gated using Flow Jo software (Tree Star Inc., Ashland, OR, USA). The Th17 analysis was done according to previously published protocol (40), in brief, PBMC was incubated for at least 1 h after thawing in media containing RPMI1640, 10% FBS, 1% pen strep, 1% l-glutamine. Samples were activated with soluble function grade anti-CD3 (OKT3 1.0 µg/ml; eBioscience, San Diego, CA, USA), soluble anti-CD28 (CD28.2; 1.0 µg/ml; eBioscience), neutralizing anti-IFN-γ and anti-IL-4 antibodies (5 µg/ml, R&D systems, Minneapolis, MN, USA), and 10 ng/ml of recombinant human IL-23 (R&D systems). The Th17 proportion was determined with CD3 and CD4 surface staining, followed by intracellular cytokine staining: samples were incubated for 4 h with ionomycin, phorbol myristate acetate, and brefeldin A, stained for CD3 (UCHT-1), and CD4 (RPA-T4), then fixed and permeabilized with a BD Cytofix/Cytoperm kit (BD Biosciences), followed by staining for IFN-γ (B27) and IL-17A (SCPL1362) antibodies from BD Biosciences. The proportion of Th17 cells was the percentage of cells with the phenotype CD3^+^CD4^−^IL-17A^+^IFN-γ^−^. The Th17 change was calculated by dividing the proportion after therapy by the proportion before therapy and multiplying by 100%. For the CD56-depletion, samples were thawed and incubated in media for at least 1 h. The samples were divided, half kept complete, the other half depleted with CD56 positive selection by magnetic activated cell sorting according to the manufacturer’s instructions (Miltenyi Biotec, Auburn, CA, USA). Depletion was confirmed with flow cytometry using antibodies for CD3 (UCHT-1) and CD56 (B159). Complete or depleted samples were then incubated with functional grade anti-CD3 (OKT3 1.0 µg/ml; eBioscience), anti-CD28 (CD28.2; 1.0 µg/ml; eBioscience), and neutralizing anti-IFN-γ and anti-IL-4 antibodies (5 µg/ml, R&D systems) for 4 days, then intracellular cytokine stained for IL-17A and IFN-γ, and supernatants analyzed for IL-17A using uncoated enzyme-linked immunosorbent assay (ELISA) kit for human IL-17A (eBioscience) and human IFN-γ (eBioscience).

### Mechanism of Action of Cytotoxicity Between NK Cells and Th17 Cell in Healthy Human Samples

PBMCs were isolated from venous blood of healthy adult participants following informed consent per approved institutional protocol (McGill University). CD4 memory T cells were obtained by magnetic activated cell sorting with negative selection, which depletes CD45RA, CD8, CD14, CD16, CD19, CD56, CD36, CD123, anti-TCRγδ, and CD235a (glycophorin A) according to manufacturer’s instructions (Miltenyi Biotec, Auburn, CA, USA). The resulting phenotype was CD3^+^CD4^+^CD45RO^+^CD45RA^−^ as determined by flow cytometry with antibodies for CD3-PerCP (UCHT-1), CD4-FITC (RPA-T4), CD45RO-APC (UCHL1), and CD45RA-PE (HI100) from BD Biosciences. Purified autologous NK cells were obtained with CD56^+^ selection magnetic activated cell sorting (Miltenyi Biotec). The resulting phenotype was CD3^−^CD56^+^ determined by flow cytometry using antibodies for CD3-FITC (UCHT-1), NKG2D-APC (1D11), and CD56-PE (B159). The purified CD4 T cells and NK cells were incubated in media containing RPMI1640, 10% FBS, 1% pen strep, 1% l-glutamine, at 37°C with 5% CO_2_, in 96-well U-bottom plates at 0.2 × 10^6^ cells per well. Activation was with soluble anti-CD3 antibodies (OKT3; 1.0 µg/ml; eBioscience) and anti-CD28 antibodies (CD28.2; 1.0 µg/ml; eBioscience). Th17 polarization was with neutralizing anti-IFN-γ and anti-IL-4 antibodies (5 µg/ml, R&D systems) and 10 ng/ml of recombinant human IL-23 (R&D systems) for 4 days. ELISA and intracellular cytokine staining were performed as previously described to measure IL-17A and IFN-γ (40). Detection of MICA was achieved with MICA polyclonal antibody and Zenon labeling kit according to manufacturer’s instructions (Thermo Fisher Scientific, Carlsbad, CA, USA). For proliferation assay, memory CD4 T cells were pre-labeled with CFSE as previously described (40). For cell death studies, annexin V and 7AAD were performed on live cells, this was done by washing cells, adding annexin V PE in annexin V binding buffer (BD Bioscience) at 1:10 ratio in 100 µl for 20 min, and then 7AAD (BD Bioscience) at a 1:20 ratio for additional 10 min at room temperature before reading on flow cytometer within 1 h. Before annexin V and 7AAD labeling, surface staining with CD4 and CD56 was performed to identify gates using FlowJo software (Tree Star Inc.). The NKG2D neutralizing antibody (Amgen, Inc., Seattle, WA, USA) was used at 40 µg/ml, an amount shown previously to significantly attenuate cytotoxicity of NK cells toward glial cells as described in a previous publication (34). CD58 neutralizing antibodies and respective isotype control were used at 10 µg/ml (R&D systems). These neutralizing experiments were done by combining purified CD4 memory T cells and purified NK cells at equal ratio and incubating for 4 days under activation (CD3 and CD28) and polarization conditions (anti-IFN-γ and anti-IL-4), then measuring IL-17A by ELISA from the supernatants.

### RNA Extraction and PCR

Quantitative PCR was performed as previously described (43, 44). In brief, total RNA was extracted using RNeasy^®^ Mini kit (QIAGEN, Toronto, ON, Canada) and then transcribed into complementary DNA using QuantiTect^®^ Reverse Transcription kit (QIAGEN) according to the manufacturer’s instructions. Relative gene expression levels were determined using primers and TaqMan^®^ FAM™-labeled MGB probes for ROR-γ, IL17, and ribosomal 18 S (VIC^®^-labeled probe) (Applied Biosystems, Foster City, CA, USA). Quantitative PCR cycling was performed for 40 cycles in a 7900HT fast-real-time PCR system (Applied Biosystems) as recommended by the manufacturer instructions. Gene-specific messenger RNA was normalized compared with 18S, the endogenous control.

### Statistics

Statistical comparison with multiple comparisons was made using one-way ANOVA (*p* < 0.05), followed by *post hoc* Tukey’s honest significant difference test. For statistical comparison of two groups before and after aHSCT a paired Student’s *t*-test was used. The level of significance is indicated by *p < 0.05 was considered to be significantly different, where indicated, **p < 0.01, and ***p < 0.001. Unless otherwise indicated, the error bars represent SEM.

## Results

### NK Cells Regulate Th Cell Responses After aHSCT in MS

**Figure 1:**
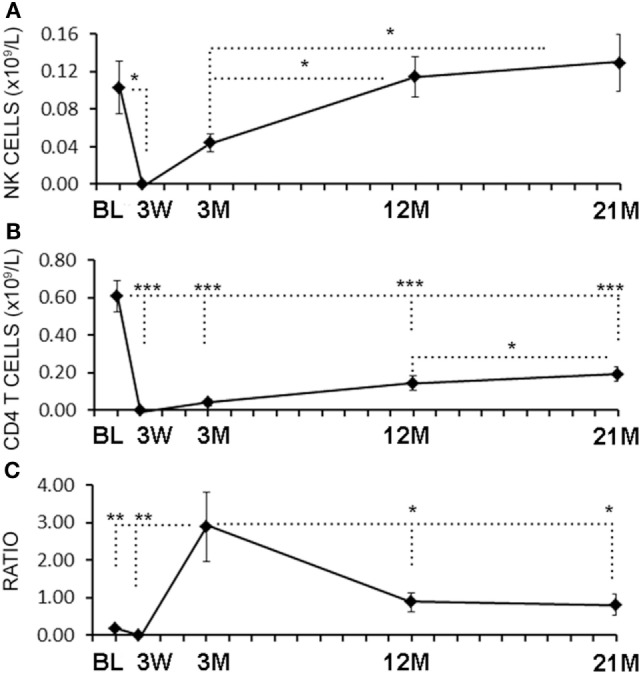
Rapid reconstitution kinetics of natural killer (NK) cell counts compared with CD4 T cells in aHSCT-treated multiple sclerosis (MS) patient blood. From freshly drawn blood samples, absolute number of NK cell counts **(A)** and CD4^+^ T cell counts **(B)** were determined at baseline (BL), and at 3 weeks (3 W), 3 months (3 M), 12 months (12 M), and 21 months (21 M) post-aHSCT. The ratio of NK cells to CD4^+^ T cells was calculated **(C)**. *N* = 7 patients.

**Figure 2:**
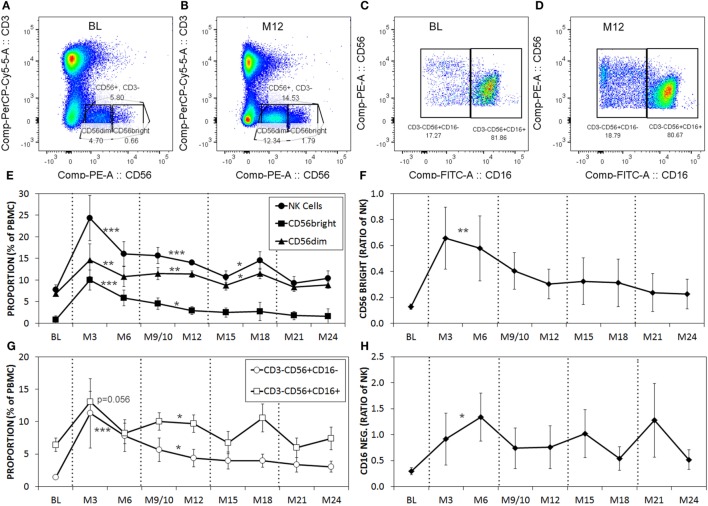
The proportions of CD56^dim^ and CD56^bright^ natural killer (NK) cells are higher post-aHSCT compared witho baseline (BL). Cryopreserved peripheral blood mononuclear cell (PBMC) from aHSCT-treated MS patients was analyzed by flow cytometry. Representative plots for BL **(A,C)**, and month 12 [M12; **(B,D)**] stained for CD3 and CD56 **(A,B)**, gated on CD3^−^CD56^+^, then shown as CD56 with CD16 **(C,D)**. Example gates for CD56^bright^ and CD56^dim^ are shown. Additional time points include BL, and serial samples approximately every 3 months for up to 24 months post-aHSCT. The average proportions of total NK cells as well as CD56^dim^ and CD56^bright^ NK cell subsets expressed as a proportion of total PBMC **(E)**. The ratio of NK cells with the CD56^bright^ phenotype calculated as CD56^bright^/CD56^dim^ NK cells **(F)**. The proportions of NK cells with CD16^+^ or CD16^−^ phenotype expressed as a proportion of total PBMC **(G)**. The ratio of gated NK cells with the CD16^−^ phenotype calculated as CD16^−^/CD16^+^ **(H)**. *N* = 7 patients. For statistical analysis, the time points were grouped in M3–M6, M9–M12, M15–M18, and M21–M24, followed by univariate one-way ANOVA with pairwise comparisons with the BL values.

Serial blood samples were collected before aHSCT, 3 weeks after treatment and then at approximately 3-month intervals thereafter. The number of NK cells began returning toward baseline (BL) levels by 3 months post-ablation, whereas CD4^+^ T cell counts did not reach BL levels even at 21 months (Figures 1A,B). The relatively rapid return of NK cells during immune-reconstitution may have been due to insensitivity to the chemotherapy or that NK cells or NK cell precursors were present in the graft. The ratio of circulating NK cells to CD4^+^ T cells increased from less than 0.2:1 before aHSCT, to a high of 3:1 at 3 months post-aHSCT, and then a steady-state ratio of 1:1 from 12 months post-aHSCT onward (Figure 1C). A more detailed analysis of immune cell subsets revealed that frequencies of both the CD56^dim^ and the CD56^bright^ NK cell subsets rose sharply between month 3 and month 6 post-aHSCT, and remained elevated until month 18 and were still significantly higher at 12–18 months post-aHSCT samples (Figures 2A,B,E). The ratio of NK bright cells (CD3^−^CD56^bright^/CD3^−^CD56^dim^) was approximately 0.1 at BL, significantly increased to a ratio of 0.6 from month 3 to 6, and dropped to BL values by 24 months (Figure 2F). We also used CD16 as a defining marker for NK bright and dim cells (Figures 2C,D). The CD3^−^CD56^+^CD16^−^ NK cells were significantly higher at month 3 until month 12 compared with BL, as were the CD3-CD56^+^CD16^+^ NK cells although they only has a trend to increase from month 3 to month 6 (Figure 2G). The ratio of CD3^−^CD56^+^CD16^−^ cells (CD3^−^CD56^+^CD16^−^/CD3^−^CD56^+^CD16^+^) was approximately 0.3 at BL, and this ratio rose significantly to about 1.0 from month 3 to month 6, and then declined to BL levels by month 24 (Figure 2H). Thus, we found similar conclusions whether the CD56 intensity or the CD16 expression was used to define NK bright and dim cells.

**Figure 3:**
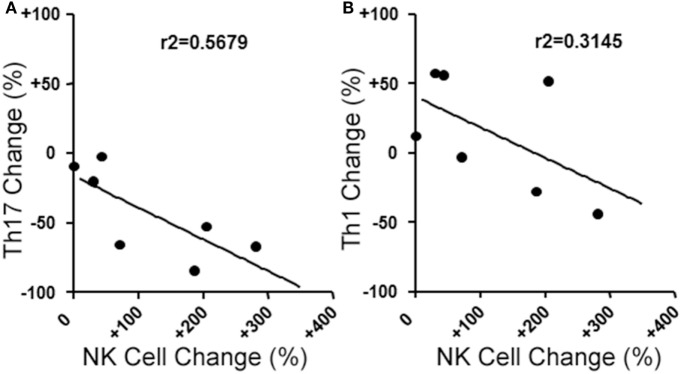
Changes in blood natural killer (NK) cell populations correlate with changes in helper T (Th) cells populations following aHSCT in MS patients. MS patient peripheral blood mononuclear cell (PBMC) samples from baseline (BL) and 12 month post-aHSCT were activated *in vitro* with anti-CD3, anti-CD28, and Th17 polarizing factors for 4 days. Th17 and Th1 cells were assessed by analysis of cytokine production by intracellular flow cytometry (CD3^+^CD4^+^IL-17A^+^IFN-γ^−^ or CD3^+^CD4^+^IL-17A^−^IFN-γ^+^, respectively). The change in frequency of Th17 cells **(A)** or Th1 cells **(B)** was plotted against the change in NK cell frequency, and linear regression was performed on the data points. *N* = 7 patients.

**Figure 4:**
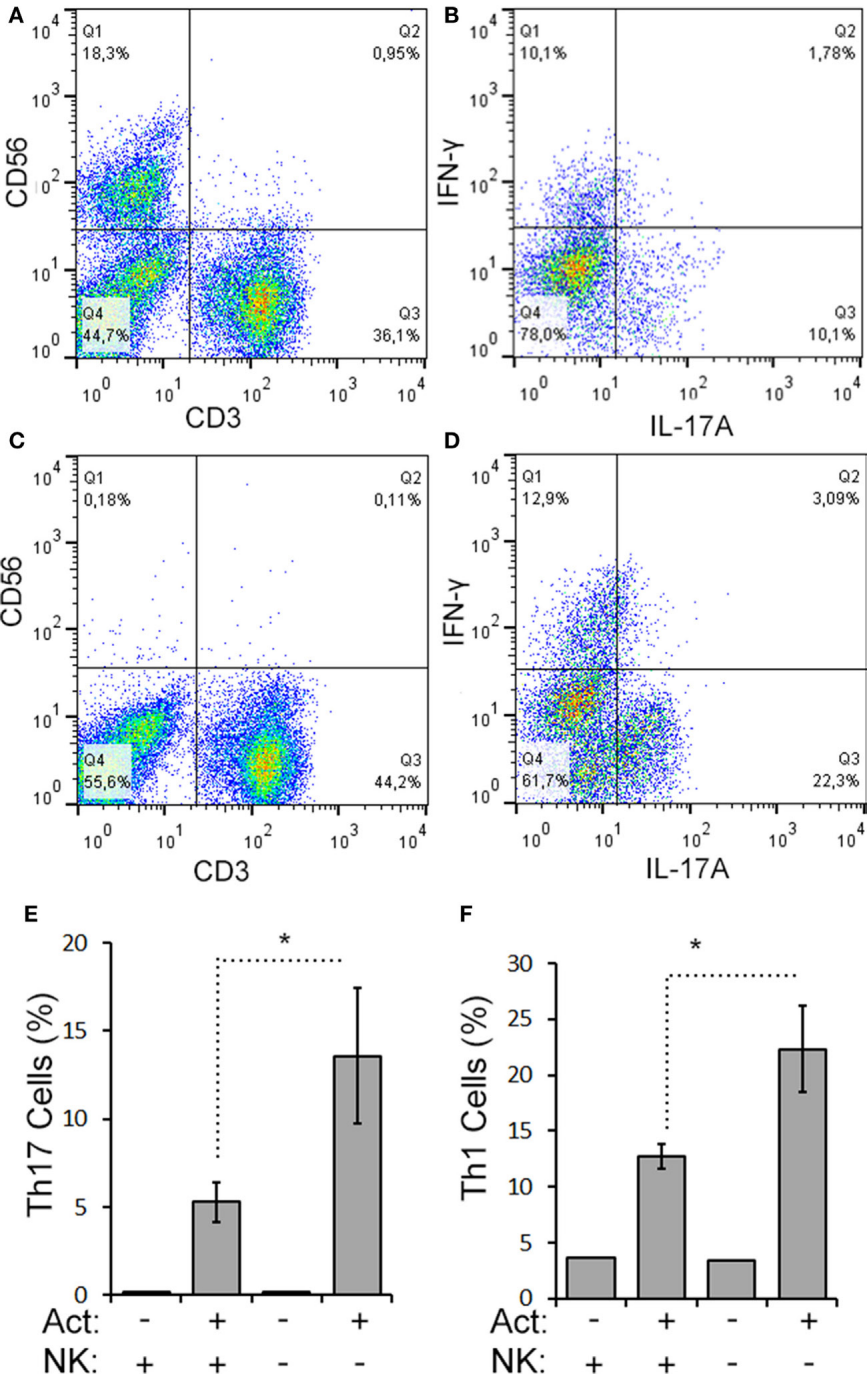
CD56^+^ cells suppress Th17 and Th1 cell responses in aHSCT. CD56^+^ cells were depleted from MS patient PBMC samples collected at 12 months post-aHSCT. Representative plots are shown for complete **(A,B)** and CD56-depleted samples **(C,D)**. Cells were activated *in vitro* with anti-CD3, anti-CD28, and Th17 polarizing factors (Act) for 4 days. The proportions of Th17 and Th1 cells were assessed by analysis of cytokine production by intracellular flow cytometry. Representative plots are shown for complete samples **(B)** and CD56-depleted samples **(D)**. The average proportion of Th17 **(E)** or Th1 cells **(F)** is shown. *N* = 3 patients.

We next evaluated the relationship between the level of change of NK cells and Th17 responses in the aHSCT samples. The proportional change in Th17 cells was inversely correlated to the change in NK cells in PBMC samples. Patients with a greater increase in NK cells exhibited the greatest reductions in Th17 responses (*r*^2^ = 0.5679; Figure 3A). Within the Th17-polarized cultures, there was a substantial proportion of CD3^+^CD4^+^IL-17A^−^ IFN^+^ cells consistent with Th1 cells. The proportion of Th1 cells was variable, with no overall reduction following aHSCT, and the change in Th1 cells correlated to changes in NK cells, but to a lesser degree than Th17 cells (Figure 3B). We next sought to determine the effect of NK cells on the induction of T cell responses *ex vivo*. To do this, CD56^+^ cells were depleted from post-aHSCT PBMC samples, which successfully removed NK cells (Figures 4A,C). This procedure also removed CD3^+^CD56^+^ cells, which was a minor population in the post-aHSCT samples (Figure 2B). The proportion of Th17 cells and Th1 cells was significantly lower in activated PBMC that contained CD56^+^ cells, compared with CD56-depleted cultures (Figure 4). On average, Th17 and Th1 cells were 2.6 and 1.8-fold higher in the absence of CD56^+^ cells, respectively, suggesting that Th17 cells were more sensitive to the presence of NK cells. Thus, NK cells may contribute to the regulation of T cell subset responses following aHSCT therapy in MS patients.

### NK Cells Kill Th17 Cells by NKG2D-Mediated Necrosis

**Figure 5:**
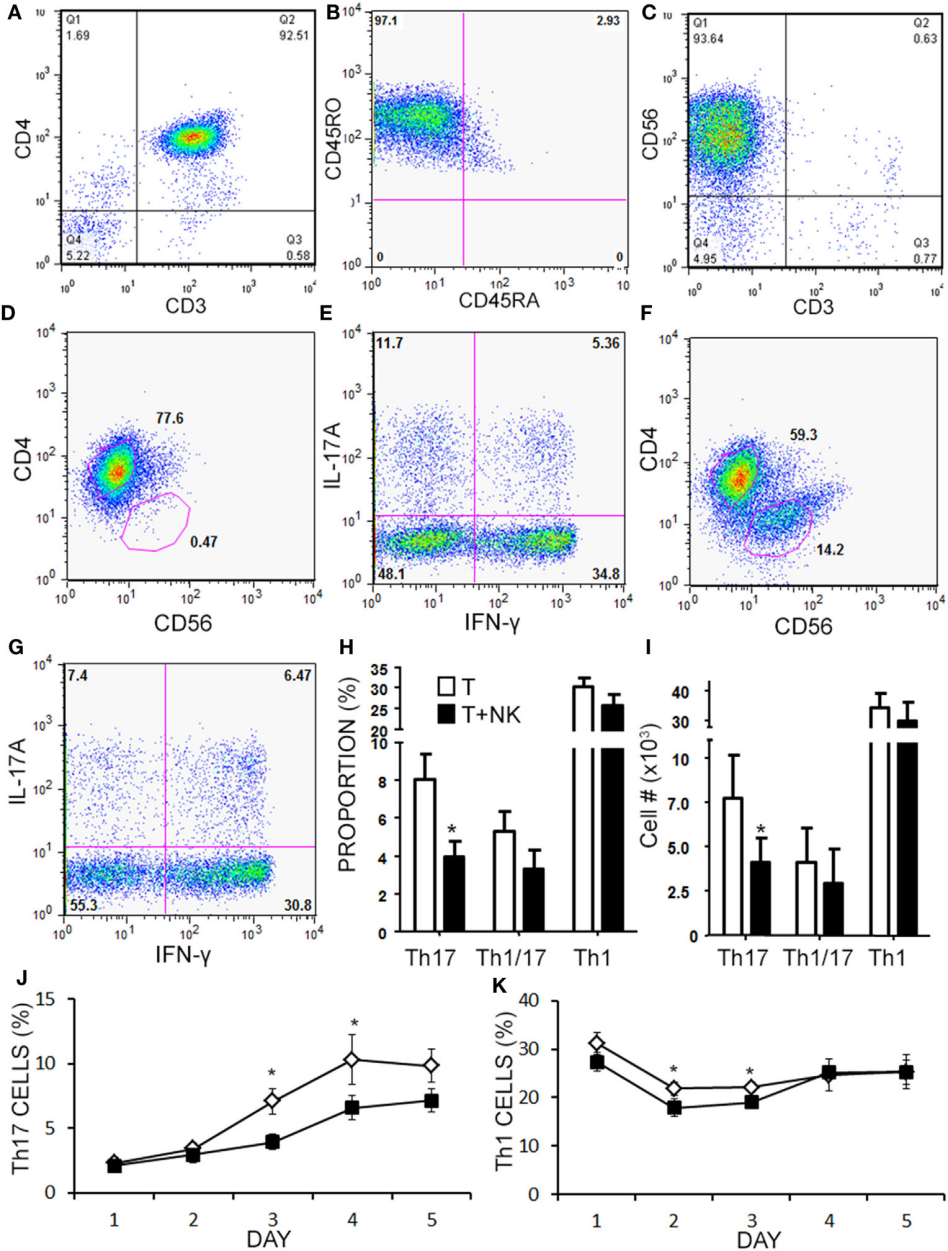
Natural killer (NK) cells reduce the proportion of Th17 and Th1 cells *in vitro*. Memory CD4^+^ T cells and NK cells were isolated from healthy subject PBMC. Memory CD4^+^ T cells were CD3^+^CD4^+^CD45RO^+^CD45RA^−^ as shown in representative dot plots **(A,B)**. NK cells were CD3^−^CD56^+^ as shown in a representative dot plot **(C)**. Memory CD4^+^ T cells were cultured without **(D,E)**, or with NK cells at a 1:1 ratio **(F,G)** for 4 days with anti-CD3, anti-CD28, and Th17 polarizing factors for 4 days. Plots are shown for CD4 × CD56, which was used to gate CD4^+^ helper T (Th) cells. Expression of IL-17 and IFN-γ by CD4^+^ T cells was assessed by intracellular flow cytometry (Th17 = CD3^+^CD4^+^IL-17A^+^IFN-γ^−^, Th1 = CD3^+^CD4^+^IL-17A^−^IFN-γ^+^, and Th1/17 = CD3^+^CD4^+^IL-17A^+^IFN-γ^+^). Data pooled from 12 experiments showing the proportion **(H)** and absolute number of Th cells **(I)**. A time-course analysis for Th17 cells **(J)** and Th1 cells **(K)** was performed for 5 days using intracellular cytokine staining. Open diamond = T cells and closed square = T cells + NK cells.

Given the limited cell numbers available from aHSCT PBMC samples, we continued to explore the mechanism in a series of cell culture experiments with healthy subjects PBMCs. Healthy PBMCs were fractioned into memory (RO^+^RA^−^) CD4^+^ T cells and CD3^−^CD56^+^ NK cells (representative purity confirmation in Figures 5A–C). Th cells and NK cells were then activated in co-culture at a 1:1 ratio to approximate the ratio observed in the post-aHSCT clinical trial samples. NK cells caused a 50% decrease in response of Th17 cells (defined as CD3^+^CD4^+^IL-17A^+^ IFN-γ^−^) when proportion and number were measured using intracellular flow cytometry (Figures 5D–I). In the same samples, the Th1/17 cells (CD3^+^CD4^+^IL-17A^+^ IFN-γ^+^) or Th1 cells (CD3^+^CD4^+^IL-17A^−^ IFN-γ^+^) were not altered by NK cells, although a trend toward decrease was noted for each (Figures 5H,I). A more detailed time-course analysis showed that NK cells began to decrease the proportion of Th17 cells on day 3, while the proportion of Th1 cells was slightly reduced on days between 2 and 3 (Figures 5J,K). Despite the fact that NK cells reduced the proportion and number of Th17 cells by about 50%, the mean fluorescent intensity of IL-17A inside of Th cells was significantly elevated from 200 MFI (SE 35) up to 283 MFI (SE 74) in the presence of NK cells (*p* = 0.042). The fluorescent intensity of IFN-γ in Th cells was not significantly altered by the presence of NK cells although a trend toward a reduction was noted, from 682 MFI (SE 85) without NK cells, to 634 MFI (SE 97) with NK cells.

**Figure 6:**
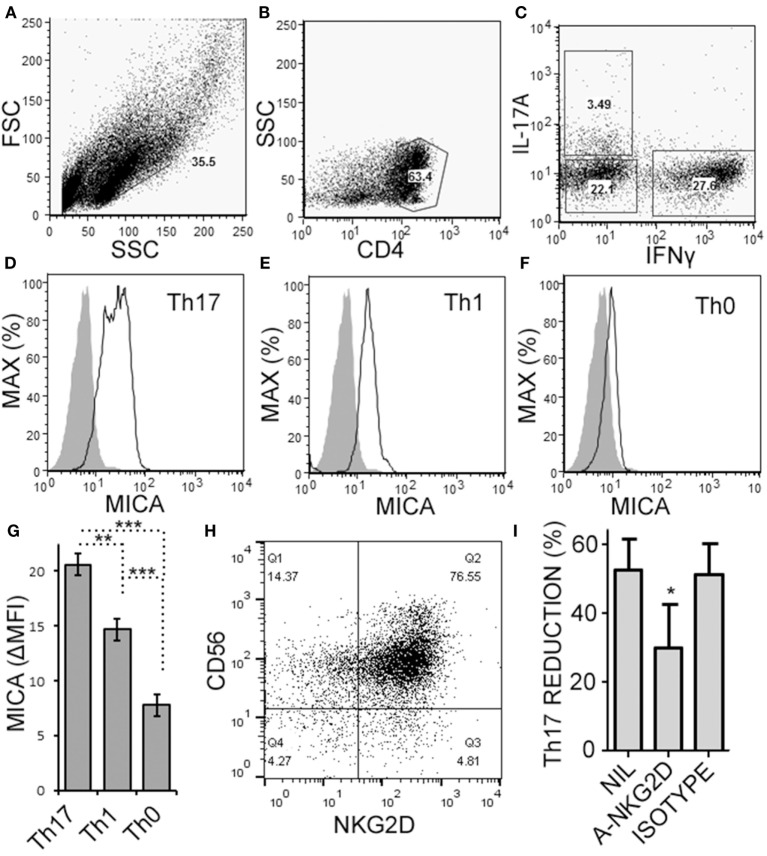
Activated memory CD4^+^ T cells express MICA and are sensitive to NKG2D-mediated natural killer (NK) cell cytotoxicity. Memory CD4^+^ T cells were obtained from healthy subject PBMC, as described in Figure 5, and activated with anti-CD3, anti-CD28, and Th17 polarizing factors for 4 days. Expression of CD4, MICA (Zenon labeled), IL-17A, and IFN-γ were assessed by intracellular cytokine staining and flow cytometry. Representative plots of FSC × SSC **(A)**, CD4 × SSC **(B)**, and IL-17A × IFN-γ **(C)** are shown. MICA expression on Th17 **(D)**, Th1 **(E)**, and the Th0 cells **(F)** is shown. Open histogram indicate MICA stained cells and closed histograms indicate an isotype control. The average mean fluorescent intensity of MICA minus the isotype control is shown [ΔMFI; **(G)**]. Expression of CD56 and NKG2D by NK cells was assessed by flow cytometry and a representative plot is shown **(H)**. NK cells were cultured with memory CD4^+^ T cells and activated with anti-CD3, anti-CD28, and Th17 polarizing factors, at the same time treated without antibody, (NIL), anti-NKG2D neutralizing antibody, or isotype control antibody **(I)**. *N* = 7 samples.

**Figure 7:**
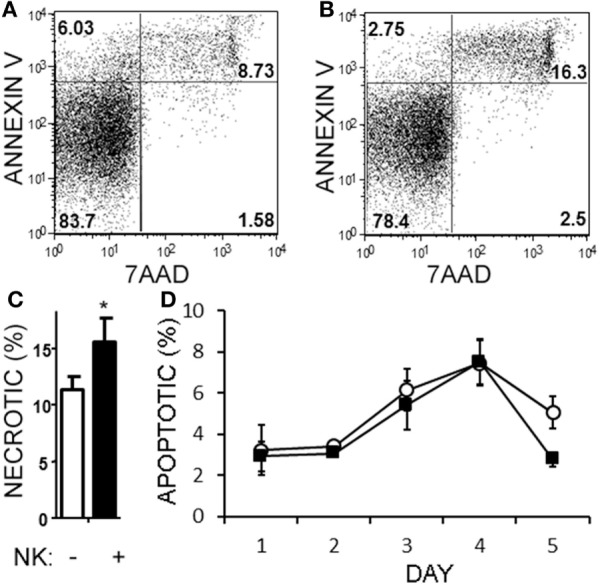
Natural killer (NK) cells are cytotoxic toward helper T (Th) cells through necrosis but not apoptosis. Memory CD4^+^ T cells from healthy subject PBMC were activated with anti-CD3, anti-CD28, and Th17 polarizing factors, either in the absence or presence of NK cells for 4 days. Samples were stained with CD3, CD56, 7AAD, and annexin V. Representative plots of T cells (CD4^+^CD56^−^) cultured without **(A)** or with NK cells **(B)** are shown. The average proportion of necrotic (7-AAD^+^annexinV^+^) T cells is shown **(C)**. Open bars = memory CD4 T cells, closed bars = memory CD4^+^ T cells plus NK cells. *N* = 8 samples. A time-course analysis of apoptotic (7-AAD^−^annexin V^+^) T cells is shown **(D)**. Open circles indicate memory CD4 T cells alone and filled squares indicate T cells plus NK cells.

Natural killer cells have previously been reported to inhibit cell cycle of T cells (45). Given the decrease in Th17 cells in the cultures over time, we questioned whether NK cells were limiting the proliferation of the T cells in our cultures. NK cells did not alter the proliferation of Th17, Th1/17, or Th1 cell as indicated by dilution of a proliferation-tracking dye combined with intracellular staining for cytokines (Figure S1 in Supplementary Material). Next, we evaluated the possibility of NKG2D-dependent cytotoxicity by NK cells. Memory CD4^+^ T cells obtained from healthy volunteer PBMCs were activated under Th17 polarizing conditions (Figures 6A–C). MICA, an NKG2D ligand, was found on Th17, Th1, and Th0 subsets (the latter defined here as negative for IL-17A and IFN-γ; Figures 6D–F), and Th17 cells had the highest MICA expression (Figure 6G). We chose to focus on MICA because the other ligands (MICB and ULBP1,2,3) were below detection thresholds on PBMC samples (data not shown). As expected, NKG2D was expressed on the NK cells and expression was identified on both CD56^dim^ and CD56^bright^ (Figure 6H). When memory CD4^+^ T cells were cultured with NK cells, neutralization of NKG2D significantly attenuated the NK cell-mediated reduction in Th17 responses (Figure 6I). To determine the mode of cell death, samples were stained with annexin V and 7-AAD dyes. NK cells caused memory CD4^+^ T cells to become annexin V^+^ and 7-AAD^+^, which is a profile consistent with late apoptosis or necrosis (Figures 7A,B). The proportion of necrotic (annexin V^+^ 7-AAD^+^) Th cells was significantly elevated after NK cells were added (Figure 7C). Furthermore, a time-course analysis revealed no evidence that NK cells were inducing apoptotic (annexin V^+^ 7AAD^−^) T cells even at earlier time points (Figure 7D). Together, these data demonstrate that NK cells can limit Th cell expansion by inducing necrotic cell death.

### CD58 Expression on NK Cells Contributes to a Transient Increase in IL-17A Production from CD4^+^ T cells

**Figure 8:**
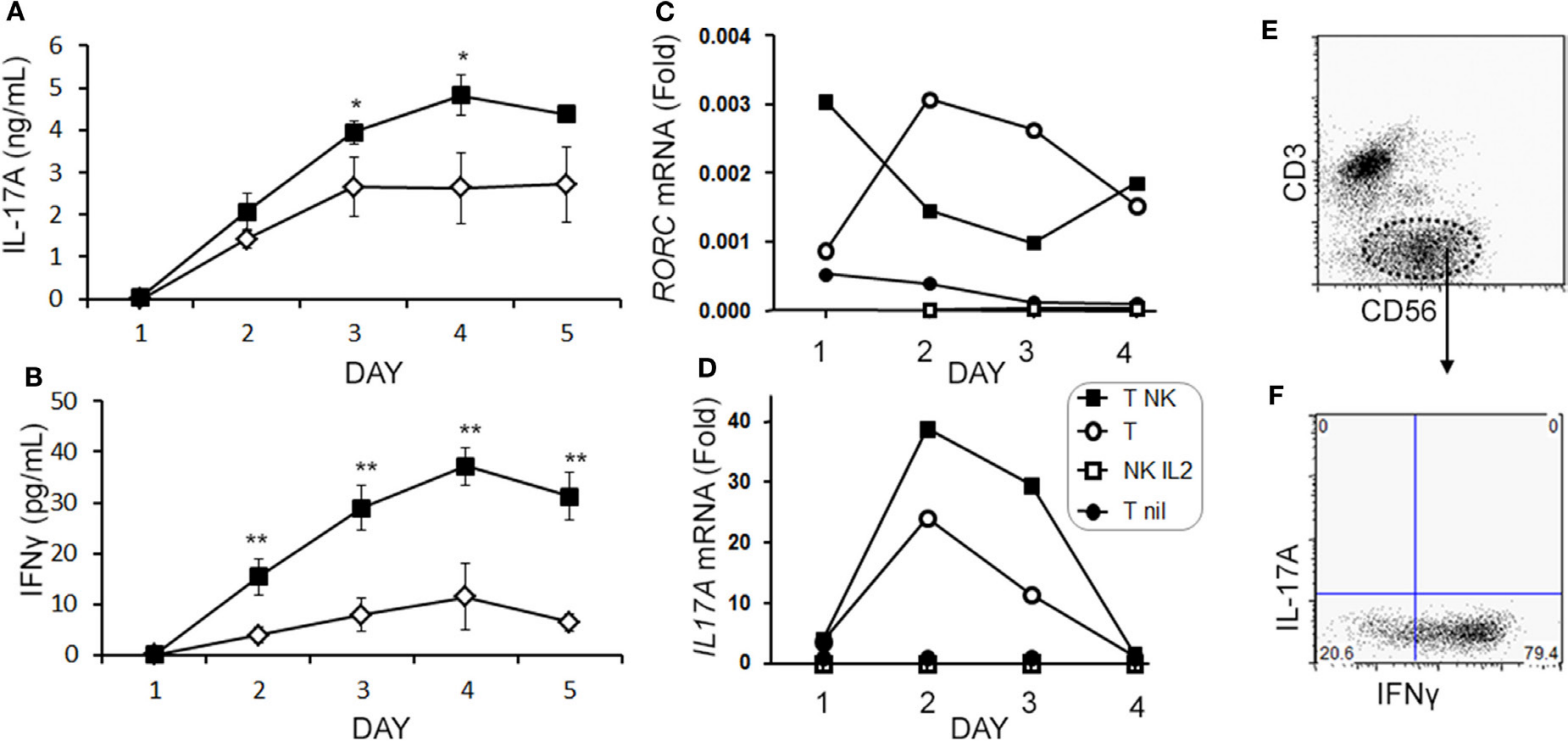
Natural killer (NK) cells augment IL-17A and *RORC* levels in memory CD4 T cells. Purified memory CD4^+^ T cells from healthy subject PBMCs were activated with anti-CD3, anti-CD28, and Th17 polarizing factors without (open diamond) or with NK cells (closed square). Cytokines IL-17A **(A)** and IFN-γ **(B)** were measured in the supernatant by enzyme-linked immunosorbent assay. Expression of *RORC* **(C)** and *IL17A* **(D)** mRNA was measured by qPCR at the indicated time points. Data are representative of three samples. Groups included non-activated memory CD4^+^ T cells (T nil; closed circles), activated memory CD4^+^ T cells (T; open circle), activated memory CD4^+^ T cells with NK cells (T NK; closed squares), and NK cells cultured alone with IL-2 (NK IL-2; open squares). Representative plots of IL-17A and IFN-γ expression in NK cells (CD3^−^CD56^+^) are shown **(E,F)**.

**Figure 9:**
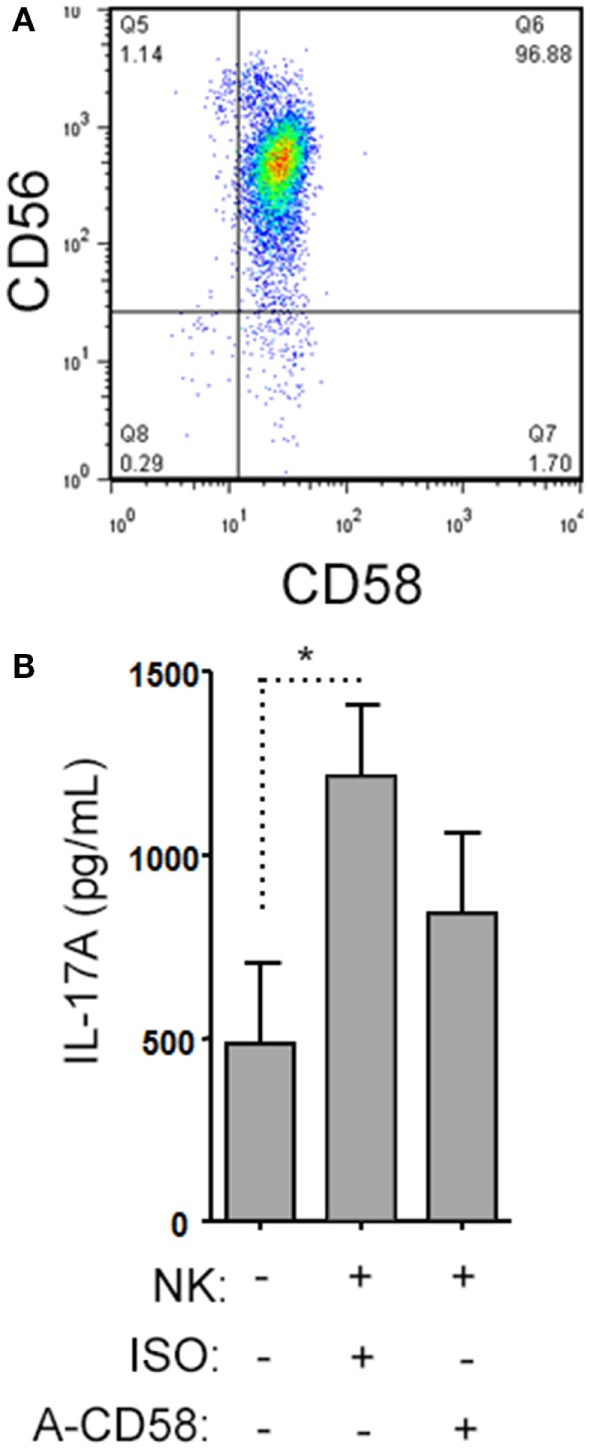
Natural killer (NK) cells support IL-17A expression by helper T (Th) cells by CD58 co-stimulation. A representative plot of CD58 expression by CD3^−^CD56^+^ NK cells is shown from healthy subject peripheral blood mononuclear cell (PBMC) **(A)**. Memory CD4^+^ T cells from healthy subjects PBMC were activated with anti-CD3, anti-CD28, and Th17 polarizing factors with NK cells in the presence or absence of CD58 neutralizing or isotype control antibody for 4 days. The cytokine IL-17A was assessed by enzyme-linked immunosorbent assay from cell culture supernatants **(B)**. Graph indicates mean IL-17A concentration for *N* = 3 samples.

**Figure 10:**
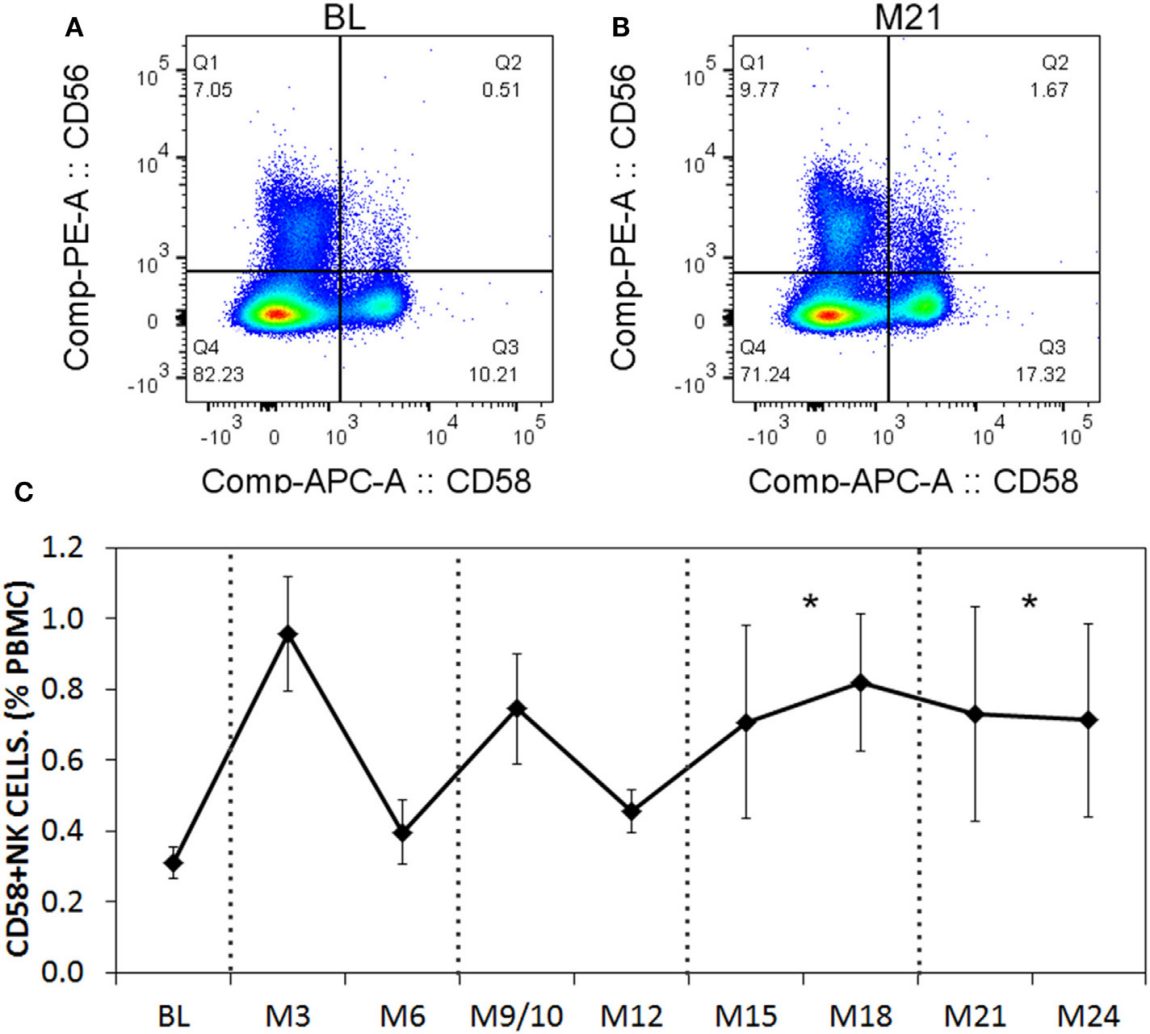
CD58 expression levels on natural killer (NK) cells before and after aHSCT treatment of multiple sclerosis (MS) patients. Cryopreserved peripheral blood mononuclear cell (PBMC) from the aHSCT cohort of MS patients was stained for CD3, CD56, and CD58. Representative plots for CD56 and CD58 are shown for baseline (BL) **(A)** and month 21 [M21; **(B)**]. A time series of samples from BL until month 24 (M24) is presented **(C)**. For statistical analysis, the time points were grouped in M3–M6, M9–M12, M15–M18, and M21–M24, followed by univariate one-way ANOVA with pairwise comparisons with the BL values. *N* = 7 patients.

When we analyzed the levels of cytokines in cell culture supernatants of activated Th cells exposed to NK cells, we observed significantly higher levels of IL-17A, and IFN-γ (Figures 8A,B). This seemed at odds with a reduced proportion of Th17 cells, however, we noted an increased MFI of IL-17A in the flow cytometry-based analysis suggesting that surviving cells were making more IL-17A. Upon activation, Th cells expressed *RORC* (encodes RORγt) mRNA on day 1 of the experiment, which increased (Figure 8C), and *IL17A* mRNA levels were detected at day 2 and day 3 of the experiment (Figure 8D). With NK cells added, there was more *RORC* on day 1, and more *IL17A* on day 2 and day 3. NK cells cultured on their own with IL-2 (a potent activator of NK cells) exhibited no detectable mRNA for either *RORC* or *IL17A*, indicating that T cells were the likely source of IL-17 in the co-cultures. Intracellular flow cytometry confirmed that the NK cells did not express IL-17A, although they did highly express IFN-γ (Figures 8E,F). One issue regarding measurement of IFN-γ is the presence of IFN-γ neutralizing antibodies used in the Th17 conditions. This lowers the amount of IFN-γ detected by ELISA when compared with cultures without the neutralizing IFN-γ (data not shown). However, the amount of IFN-γ even in the presence of the neutralizing IFN-γ was significantly increased in the presence of NK cells. To investigate the mechanism by which NK cells induced more IL-17A, selected adhesion molecules were studied. We first considered CD54 (ICAM-1), an adhesion molecule found on antigen presenting cells and NK cells. Neutralizing CD54 did not alter the NK cell-mediated transient boost in IL-17A (data not shown). CD58, another adhesion molecule, was expressed by both CD56^bright^ and CD56^dim^ NK cells (Figure 9A). The presence of neutralizing antibodies against CD58 in the co-cultures partially attenuated the NK cell-mediated increase in IL-17A (Figure 9B). To determine the expression of CD58 on NK cells from the MS cohort, we stained samples from BL until M24 and analyzed by flow cytometry (Figure 10). There was a very low percentage of CD58^+^ NK cells in these samples (less than 1% of total PBMC), this proportion was not changed at M12, and significantly higher by M15–M24 compared with BL although remaining below 1% of PBMC.

## Discussion

Autologous-HSCT is a promising new therapy for aggressive MS, which can abrogate clinical relapses and stabilize brain MRI lesions. The reconstituting immune system has a lesser neuroinflammatory capacity in post-aHSCT samples. This suggests that changes had occurred following treatment, which decrease disease progression. The data presented here demonstrate that NK cells reconstitute rapidly following aHSCT, while CD4^+^ T cells remained below BL for up to 21 months. One explanation for functional suppression of CD4 T cells could be that conventional regulatory T cells (CD3^+^ CD4^+^ FoxP3^+^ CD25^+^ CD127^−^) that were shown to rapidly reconstitute following a non-ablative aHSCT in MS were suppressing CD4 T cells (46). However, in the Canadian ablative cohort, regulatory T cells were only transiently increased in MS patients after aHSCT, and returned to BL by 9 months post-treatment (40). Another possibility is that thymic atrophy accounts for the low Th cell counts. However, the re-emergence of recent thymic emigrant naive T cells, and the broad clonal diversity that developed in MS patients who underwent aHSCT, suggests that the thymus (which exhibits decreased function early after chemotherapy) is still sufficient to facilitate T cell maturation and selection (40, 47). In this study, we tested the hypothesis that NK cells were playing a regulatory role in the reconstituting immune system. NK cells correlated inversely with the change in Th17 cells, and depleting NK cells from aHSCT samples resulted in a higher proportion of Th17 cells. Th1 cells were also inhibited by NK cells, albeit to a lesser extent than Th17 cells. These results indicate that NK cells reconstitute rapidly, possibly due to incomplete ablation or presence of NK cells in the graft, following aHSCT and suppress the re-emergence of Th17 cells.

Natural killer cells are known to suppress T cells, but the relative effect on Th cell subsets has not been widely explored. In cell culture experiments, we found that NK cells reduced the proportion of Th17 cells by necrosis in an NKG2D-dependent fashion. Previous studies have reported that NK cell–T cell interactions may result in apoptosis (48), or limit proliferation of T cells (45). Here, we only observed necrotic cell death of T cells with no differences in apoptosis or proliferation of Th cells. Th17 cells expressed more MICA than Th1 cells that may account for the higher sensitivity of Th17 cells to NK cell-mediated cytotoxicity. Despite the clear necrotic effects, NK cells caused an early rise in expression of IL-17A and RORγt. Moreover, the IL-17A increase was, in part, mediated by CD58. Thus, NK cells provide early co-stimulation to Th17 cells followed by NKG2D-dependent cytotoxicity. We previously identified a decrease in IL-17A in culture supernatants of PBMC (activated with anti-CD3, anti-CD28, and Th17 polarizing factors) at 12 months post-aHSCT (40). While it is possible that NK cells transiently boosted Th17 responses, the longer-term outcome is that Th17 responses, whether assessed by flow cytometry or ELISA, were reduced in post-aHSCT samples. Further examination of the aHSCT MS samples showed a very low level (<1%) of CD58^+^ NK cells, and this value increased slightly by month 15 post therapy. It is not clear that this has a biologically significant impact on the patients.

Our observations add to the growing understanding of how NK cells regulate T cell responses. NK cells can provide co-stimulation and even antigen presentation that activates T cells (49, 50). Upon activation, T cells upregulate NKG2D ligands and are then reduced in number through cytotoxic action of NK cells (26–28). We did not perform kinetics of expression of MICA, however, a previous study showed that T cells only express MICA and related ligands after 3 days of activation, leading to their sensitization to NK cell-mediated killing (27). This timing is consistent with our observations where NK cells were initially costimulatory, and then began to kill by necrosis by the third day of the cell culture experiment. Thus, there is a brief window of time for NK cells to boost Th17 cell responses. In our experiments, the proportion of Th17 cells remaining after NK cell exposure was around 50% which suggests that the purpose of NK cells is to attenuate, but perhaps not fully eliminate, T cell expansion, to preserve immunological memory while preventing uncontrolled T cell activation. This may allow NK cells to accelerate the normal life cycle of Th17 cells by transiently boosting IL-17A expression and secretion, and then accelerating down-regulation of the effector response through cytotoxicity.

We identified an increase in both CD56^bright^ and CD56^dim^ NK cells at 12 months post-aHSCT. Similar conclusions were obtained whether the CD56 intensity or the CD16 expression was used to define NK cell subsets. The ratio of CD56^bright^ NK cells was substantially increased from month 3 to month 6 after the therapy suggesting that they replenished faster than the CD56^dim^ NK cells. From our phenotype data it is not possible to draw conclusions about the functional properties of NK cell subsets. In an elegant study by Laroni et al., the two subsets were sorted from healthy donors and their cytotoxic capacity was compared in the presence of inflammatory cytokines (25). The authors found that CD56^bright^ NK cells were more cytotoxic, and they required pro-inflammatory cytokines to be cytotoxic to human autologous CD4 T cells. However, other studies found comparable cytotoxicity of CD56^bright^ and CD56^dim^ NK cells toward human autologous CD4 T cells (51). Thus, both subsets can have cytotoxic capacity depending on the protocols used. The ability for NK cells to potentially up- or down-regulate inflammatory T cell responses warrants further detailed investigation into the molecular mechanisms of NK cell-T cell interactions, and into their clinical relevance for novel therapeutic strategies.

## Canadian MS/BMT Study Group

The Canadian MS/BMT Study Group includes **Harold Atkins**, **Lothar Huebsch**, **Lisa Walker**, **Marjorie Bowman**, **Mark S. Freedman**, Ottawa Hospital Research Institute (OHRI), Ottawa, ON, Canada; **Amit Bar-Or**, **David Haegert**, **Douglas Arnold**, **Farzaneh Jalili**, **Hyunwoo Lee**, **Jack Antel**, **Jacqueline Chen**, **Peter Darlington**, **Pierre Laneuville**, **Robert Brown**, **Yves Lapierre**, Montreal Neurological Institute/McGill University, Montreal, QC, Canada; **Rafick Pierre Sekaly**, **Remi Cheynier**, Université de Montreal, Montreal, QC, Canada; **Paul O’Connor**, University of Toronto, Toronto, ON, Canada.

## Ethics Statement

Samples were collected, as specified in the immune monitoring sub study, from patients enrolled in the trial “Autologous Stem Cell Transplant for Multiple Sclerosis (MS/BMT)” (NCT01099930) following informed consent as approved by the institutional review boards. PBMCs were isolated from healthy donors following informed consent per approved institutional protocol (McGill University).

## Author Contributions

PD designed, performed, analyzed experiments as well as made figures, wrote and edited the manuscript. BS made figures, wrote and edited the manuscript. TT conceived of project, designed performed, analyzed, experiments. J-SD performed and analyzed experiments. LF conceived project and designed experiments. MR designed performed and analyzed experiments. M-NB designed, performed and analyzed experiments as well as made figures. NA designed performed and analyzed experiments as well as edited the manuscript. MF designed implemented transplant procedure, analyzed blood samples and edited the manuscript. HA designed implemented transplant procedure, analyzed blood samples, and edited the manuscript. AB-O conceived project, designed and analyzed experiments, analyzed clinical data, wrote and edited the manuscript.

## Conflict of Interest Statement

The authors declare that the research was conducted in the absence of any commercial or financial relationships that could be construed as a potential conflict of interest.

## Supplementary Material

Figure S1 Natural killer (NK) cells do not inhibit helper T (Th) cell proliferation. Memory T cells from healthy subjects were labeled with CFSE and then activated with anti-CD3, anti-CD28, and Th17 polarizing factors with NK cells for 4 days. T cell proliferation was determined by CFSE dilution as assessed by flow cytometry. Th cell subsets were identified based on cytokine production by intracellular flow cytometry. Representative histograms indicating CFSE dilution are shown for Th17 **(A)**, Th1/17 **(B)**, and Th1 **(C)** cells. Non-activated T cells (gray filled), activated T cells (thick line), and activated T cells cultured with NK cells (thin line) are shown. The average proportion of T cells that have undergone division are shown **(D)**. Open bars = T cells, closed bars = T cells plus NK cells. *N* = 7 samples.
