# Supplementary figures and images for: Natural Killer Cells Regulate Th17 Cells After Autologous Hematopoietic Stem Cell Transplantation for Relapsing Remitting Multiple Sclerosis
Source: Front Immunol. 2018 May 7;9:834. doi: 10.3389/fimmu.2018.00834 (PMC5951114; doi:10.3389/fimmu.2018.00834)

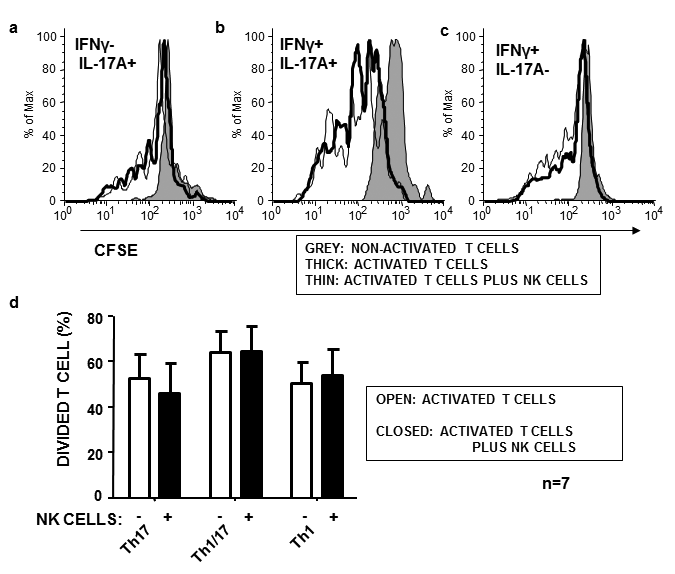

Supplement: Figure S1 — Natural killer (NK) cells do not inhibit helper T (Th) cell proliferation. Memory T cells from healthy subjects were labeled with CFSE and then activated with anti-CD3, anti-CD28, and Th17 polarizing factors with NK cells for 4 days. T cell proliferation was determined by CFSE dilution as assessed by flow cytometry. Th cell subsets were identified based on cytokine production by intracellular flow cytometry. Representative histograms indicating CFSE dilution are shown for Th17 (A), Th1/17 (B), and Th1 (C) cells. Non-activated T cells (gray filled), activated T cells (thick line), and activated T cells cultured with NK cells (thin line) are shown. The average proportion of T cells that have undergone division are shown (D). Open bars = T cells, closed bars = T cells plus NK cells. N = 7 samples. [file image_1.tif]
